# An online method to monitor hand muscle tone during robot-assisted rehabilitation

**DOI:** 10.3389/frobt.2023.1093124

**Published:** 2023-02-06

**Authors:** Raffaele Ranzani, Giorgia Chiriatti, Anne Schwarz, Giada Devittori, Roger Gassert, Olivier Lambercy

**Affiliations:** ^1^ Rehabilitation Engineering Laboratory, Department of Health Sciences and Technology, ETH Zurich, Zurich, Switzerland; ^2^ Department of Industrial Engineering and Mathematical Science, Polytechnic University of Marche, Ancona, Italy; ^3^ Vascular Neurology and Neurorehabilitation, Department of Neurology, University Hospital Zurich, University of Zurich, Zurich, Switzerland; ^4^ Future Health Technologies, Singapore—ETH Centre, Campus for Research Excellence And Technological Enterprise (CREATE), Singapore, Singapore

**Keywords:** perturbation, robot-assisted rehabilitation, hand, stroke, safety, neurorehabilitation, spasticity, muscle tone

## Abstract

**Introduction:** Robot-assisted neurorehabilitation is becoming an established method to complement conventional therapy after stroke and provide intensive therapy regimes in unsupervised settings (e.g., home rehabilitation). Intensive therapies may temporarily contribute to increasing muscle tone and spasticity, especially in stroke patients presenting tone alterations. If sustained without supervision, such an increase in muscle tone could have negative effects (e.g., functional disability, pain). We propose an online perturbation-based method that monitors finger muscle tone during unsupervised robot-assisted hand therapy exercises.

**Methods:** We used the ReHandyBot, a novel 2 degrees of freedom (DOF) haptic device to perform robot-assisted therapy exercises training hand grasping (i.e., flexion-extension of the fingers) and forearm pronosupination. The tone estimation method consisted of fast (150 ms) and slow (250 ms) 20 mm ramp-and-hold perturbations on the grasping DOF, which were applied during the exercises to stretch the finger flexors. The perturbation-induced peak force at the finger pads was used to compute tone. In this work, we evaluated the method performance in a stiffness identification experiment with springs (0.97 and 1.57 N/mm), which simulated the stiffness of a human hand, and in a pilot study with subjects with increased muscle tone after stroke and unimpaired, which performed one active sensorimotor exercise embedding the tone monitoring method.

**Results:** The method accurately estimates forces with root mean square percentage errors of 3.8% and 11.3% for the soft and stiff spring, respectively. In the pilot study, six chronic ischemic stroke patients [141.8 (56.7) months after stroke, 64.3 (9.5) years old, expressed as mean (std)] and ten unimpaired subjects [59.9 (6.1) years old] were tested without adverse events. The average reaction force at the level of the fingertip during slow and fast perturbations in the exercise were respectively 10.7 (5.6) N and 13.7 (5.6) N for the patients and 5.8 (4.2) N and 6.8 (5.1) N for the unimpaired subjects.

**Discussion:** The proposed method estimates reaction forces of physical springs accurately, and captures online increased reaction forces in persons with stroke compared to unimpaired subjects within unsupervised human-robot interactions. In the future, the identified range of muscle tone increase after stroke could be used to customize therapy for each subject and maintain safety during intensive robot-assisted rehabilitation.

## 1 Introduction

Robot-assisted rehabilitation has become an established method to complement conventional upper limb therapy after neurological injuries, such as stroke (Lambercy et al., 2018; Aggogeri et al., 2019; Demofonti et al., 2021). Several studies demonstrated the potential of robotic technologies to offer motivating task-oriented training with individualized difficulty (Metzger et al., 2014; Giang et al., 2020) and allow therapy outcomes comparable to conventional dose-matched therapies (Lo et al., 2010; Burgar et al., 2011; Mehrholz et al., 2018; Rodgers et al., 2019; Ranzani et al., 2020). One important potential of rehabilitation robots is the possibility to provide higher therapy dose, which is expected to generate higher gains (McCabe et al., 2015; Ward et al., 2019). This could be achieved by letting stroke patients accessing, in addition to conventional therapies, high-quality minimally or unsupervised robot-assisted therapy sessions, either in the clinic or at home after discharge (Sivan et al., 2014; Wolf et al., 2015; Zhang et al., 2020; Lambercy et al., 2021; Ranzani et al., 2021). However, the unsupervised use of rehabilitation devices, particularly by stroke subjects with diverse neurological deficits (e.g., sensory loss, cognitive issues), bares the risk to increase the occurrence of adverse events. Thus, a safe and comfortable interaction between the robot and the patient, and the possibility to monitor potential adverse event, are crucial to achieve effective, unsupervised robot-assisted therapy.

One of the adverse events that might occur during intensive upper limb neurorehabilitation is an increase in muscle tone. As a matter of fact, stroke survivors, particularly in the chronic stage, frequently suffer from muscle tone alterations and spasticity [i.e., between 30% and 80% (Kuo and Deshpande, 2012; Pandyan, 2005)] due to injuries in the descending motor pathways and abnormal motor drives. Muscle tone is commonly defined as the physiological continuous and passive partial contraction of the muscles, or the passive resistance of the muscles during resting state (O’Sullivan et al., 2019). Spasticity, according to the definition of (Lance, 1980), is a motor disorder that causes a speed-dependent increase in muscle tone, caused by the increased excitability of muscle spindles, as part of the upper motor neuron syndrome (Yang et al., 2021). Active motor tasks can temporarily change muscle tone and, if not properly monitored and administered, might affect the way patients engage in rehabilitation exercises and potentially influence long-term outcomes (Bobath and Bobath, 1950; Perfetti and Grimaldi, 1979; Veerbeek et al., 2017). Since uncontrolled spasticity can cause pain, muscle contractures and significantly compromise patients’ independence and quality of life (Formisano et al., 2005; Kong et al., 2012), observational assessments are typically used in clinical settings to promptly detect spasticity and monitor it over time. Two of the most widely used assessments are the Modified Ashworth Scale [MAS, (Bohannon and Smith, 1987)] and the Tardieu Scale (Tardieu et al., 1954). Unfortunately, clinical measures, and in particular the MAS, which is the most frequently used scale, lack validity and reliability, as they are subjective and rely heavily on the therapist’s experience (Katz and Rymer, 1989; Cha and Arami, 2020; Germanotta et al., 2020). Furthermore, they cannot be performed in unsupervised settings. Therefore, to complement the intrinsic safety characteristics of a rehabilitation device [e.g., electromechanical properties (Mehrholz et al., 2018; Bessler et al., 2021)], human-robot interaction could be exploited to implement monitoring strategies that study changes in patients’ motor control during rehabilitation. Robotic devices can be used to accurately estimate changes in muscle tone (or its manifestation in terms of change in joint stiffness) during limb mobilization (Artemiadis et al., 2010; Tucker et al., 2013; Dehem et al., 2017; Ranzani et al., 2019; Zou et al., 2021; van der Velden et al., 2022). Most frequently, to do this, position- or velocity-controlled perturbations are applied to the passive limb while force sensors measure the reaction force/torque of joint and muscles during the movement, which can be used to quantify muscle tone and limb biomechanics, as well as spasticity when different perturbation speeds are considered (De-la-Torre et al., 2020; Guo et al., 2022). However, to date, no device for robot-assisted therapy allows to autonomously monitor the changes in patient’s muscle tone directly during rehabilitation exercises as a way to ensure safe conditions of use during unsupervised therapy sessions. Moreover, according to a recent review, only three percent of the studies focusing on technology-assisted assessment of spasticity focus on the hand (Guo et al., 2022).

In a previous work, we proposed a method to autonomously monitor hand muscle tone parameters (i.e., end-point stiffness and forces at the fingertips) online during robot-assisted therapy exercises, and we preliminarily tested its feasibility with five unimpaired (i.e., neurologically intact) subjects and five subjects after stroke not presenting hand spasticity (Ranzani et al., 2019). In this paper, we extend and complement this work by further validating the accuracy of the method in a testbench experiment using springs instead of the human hand (i.e., the method is used to estimate the stiffness of springs with known stiffness), and by investigating the range of muscle tone fluctuations, and their dependency on the perturbation speed, in subjects with hand spasticity after chronic stroke as well as in an unimpaired age-matched population. All subjects performed a newly developed therapy exercise embedding the proposed perturbation-based method during a single therapy session. Moreover, the muscle tone parameters were compared to the MAS at the beginning and at the end of the session to evaluate their correlation. We hypothesize that the proposed method would be able to accurately estimate muscle tone/stiffness parameters and capture differences between unimpaired and stroke subjects over exercise time. In the stroke group, we also expect muscle tone parameters to be speed-dependent but not significantly correlated with the MAS due to its limited reliability and resolution (Katz and Rymer, 1989; Melendez-Calderon et al., 2013). This work could be an important step towards developing smart algorithms that automatically adapt therapy parameters to continuously ensure user’s safe use of rehabilitation robots during unsupervised therapy.

## 2 Materials and methods

### 2.1 Robotic device

**FIGURE 1:**
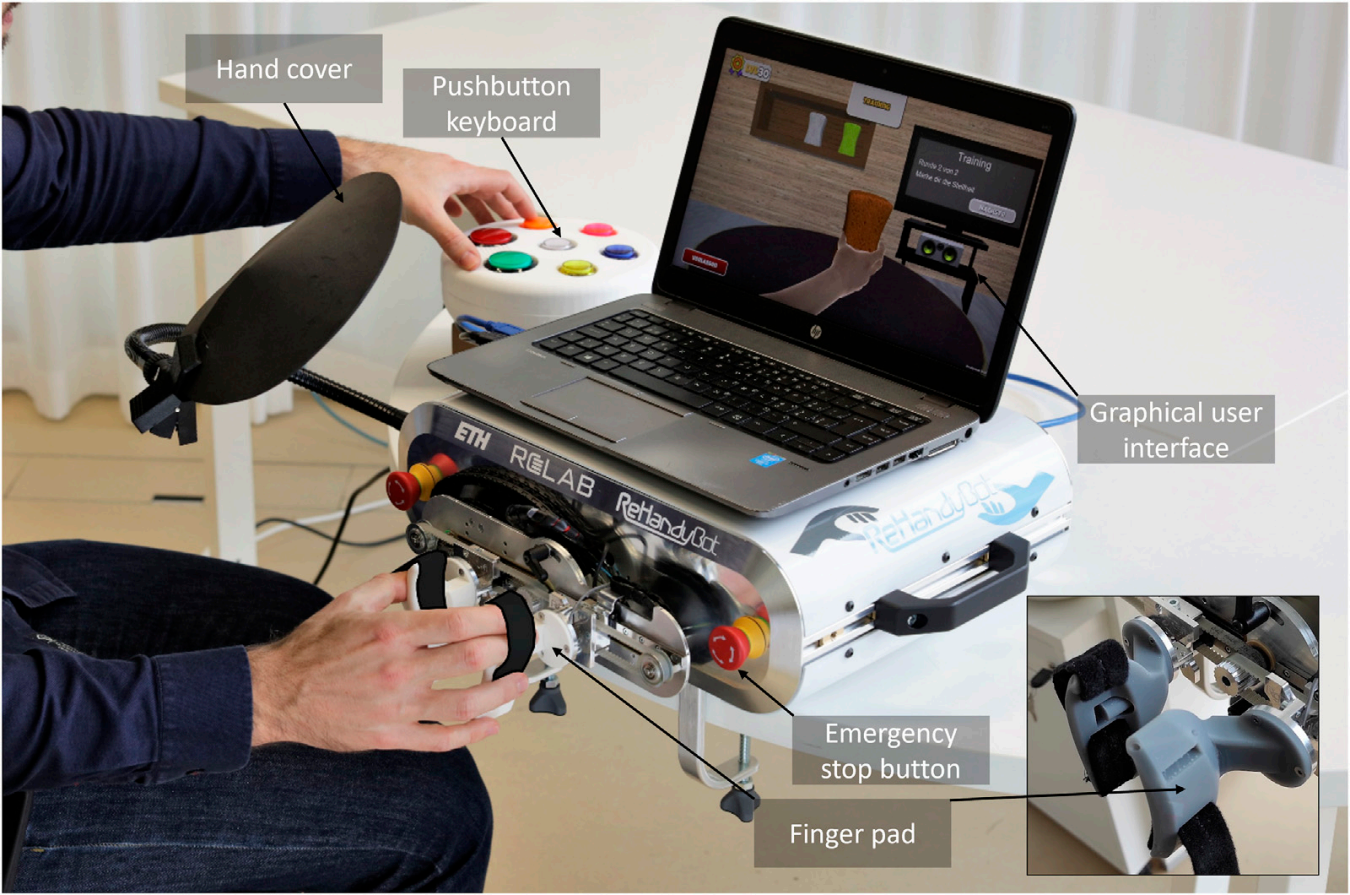
A subject performing a rehabilitation exercise (sponge exercise) with the ReHandyBot, which includes physical (i.e., instrumented finger pads, colored pushbutton keyboard) and graphical user interfaces (GUIs) and a set of therapy exercises that can be used without supervision. During the specific sponge exercise, subjects have to identify virtual sponges with different stiffness rendered by the robot by squeezing them and then press the color corresponding to the perceived stiffness on the pushbutton keyboard. A movable hand cover allows to cover the hand during the execution of the exercise. Emergency stop buttons can be pressed at any time.

The proposed study uses the ReHandyBot, a portable haptic device for hand rehabilitation, as assessment and therapy platform. We recently developed this robot as a compact and portable redesign of the ReHapticKnob (Metzger et al., 2011; Ranzani et al., 2021). It has two active degrees of freedom (DOF) that allow training of grasping (i.e., flexion-extension of the fingers) and pronosupination of the forearm during therapy exercises with haptic feedback. A virtual reality interface displays objects to interact with, in the context of engaging exercises, while their mechanical properties are rendered through instrumented finger pads held and manipulated by the users (Metzger et al., 2014). During therapy exercises, users sit in front of the robotic device with the fingers attached to its finger pads with VELCRO straps, as shown in Figure 1. The VELCRO straps constrain only the first three fingers since they are highly relevant for most grasp types used in daily life (Saudabayev et al., 2018) and their sensorimotor function is interlinked through a common innervation (of the median nerve). Moreover, this type of fixation allows to fix the hand easily and stably, independently of the hand anthropometrics, and leaves the user the freedom to keep the last two fingers flexed or extended on the finger pad surface. To allow patients with different finger muscle tone to use the device and guarantee ergonomics, the finger pads have been designed with an ellipsoidal shape. This could especially help patients with increased muscle tone to prevent fingers from sliding out of the finger pads, while allowing a simple hand placement and a comfortable grip. One-DOF load cells (Omega LCL-040 Thin Beam) are located under each finger pad and allow the measurement of the interaction forces (i.e., grasping force and pronosupination torque) between the user and the robot. The user can interact with the robot (e.g., login to his/her personalized therapy plan, select and execute the exercises) through an intuitive colored pushbutton keyboard suitable for unsupervised therapy. A movable hand cover allows to fully cover the hand during the execution of therapy exercises. The robot can easily be adapted for left and for right hand use since the finger pads are symmetrical, the hand cover can be flexibly moved and the pushbutton keyboard can be placed on the left or on the right of the robot. ReHandyBot offers the same assessments (e.g., active range of motion, aROM) and assessment-driven rehabilitation exercises previously implemented on ReHapticKnob, which aim at training grasping and forearm pronosupination movements, as well as proprioception, haptic perception, sensory-motor memory and cognitive function. More details on the therapy platform, assessments and therapy exercises can be found in (Metzger et al., 2014) and (Ranzani et al., 2021).

### 2.2 Muscle tone estimation method

**FIGURE 2:**
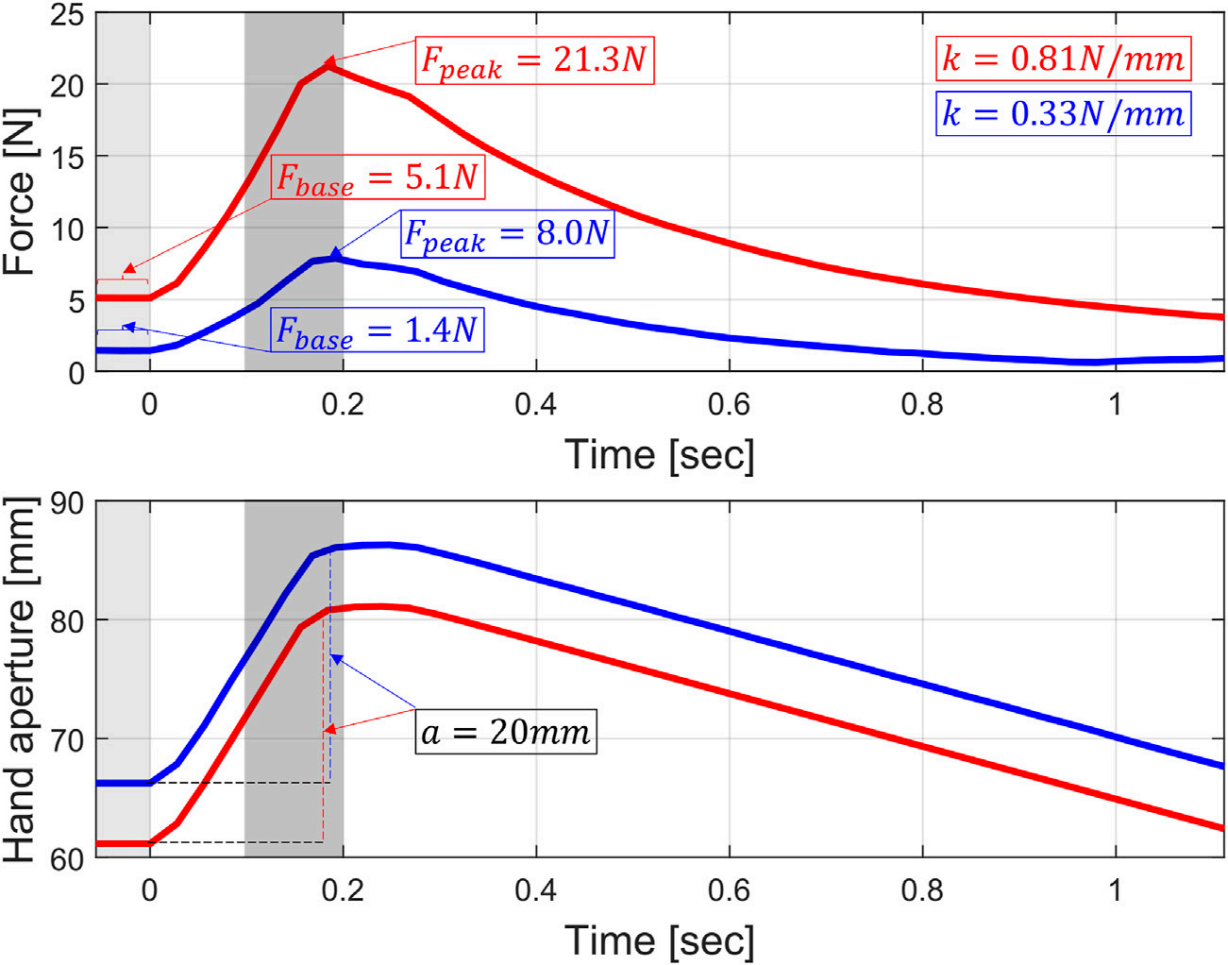
Representative fast 20 mm (amplitude *a*, thumb to index tip distance change) ramp-and-hold perturbation from subject two in the stroke group (red) and subject five in the unimpaired group (blue). In light grey is the 50 ms window in which the average baseline force (F_peak_) is calculated before the ramp onset. In grey is the 100 ms window in which the maximum force peak induced by the perturbation (F_base_) is evaluated.

Building on our previous proof of concept work (Ranzani et al., 2019), we developed and refined a muscle tone estimation method that consists of fast (150 ms) and slow (250 ms) 20 mm ramp-and-hold perturbations on the grasping DOF (i.e., 10 mm at the fingertip per finger), which can be applied during the therapy exercises to stretch the long finger flexor muscles within their range of motion, while the hand is relaxed (see example in Figure 2). Stretching of the finger flexors was chosen over stretching of the finger extensors since this is the direction that predominantly elicits spastic behavior (Katner and Kasarskis, 2014). The small perturbation amplitude at the given speed prevent overstretching of the fingers and make perturbations less perceivable, since changing subject’s awareness and stress can influence muscle tone (Boman, 1971; Burke et al., 2013; Profeta and Turvey, 2018). The chosen time windows allow to capture short (i.e., spinal monosynaptic) and long-loop (i.e., transcortical) stretch reflex reactions that are relevant for the control of muscle tone and exclude steady state voluntary reactions, which starts after approximately 750 ms (Hammond et al., 1956; Davidoff, 1992; Pruszynski et al., 2009). Two speeds allow to evaluate whether muscle tone is speed-dependent (i.e., as in the case of spasticity).

### 2.3 Therapy exercise with online muscle tone monitoring

**FIGURE 3:**
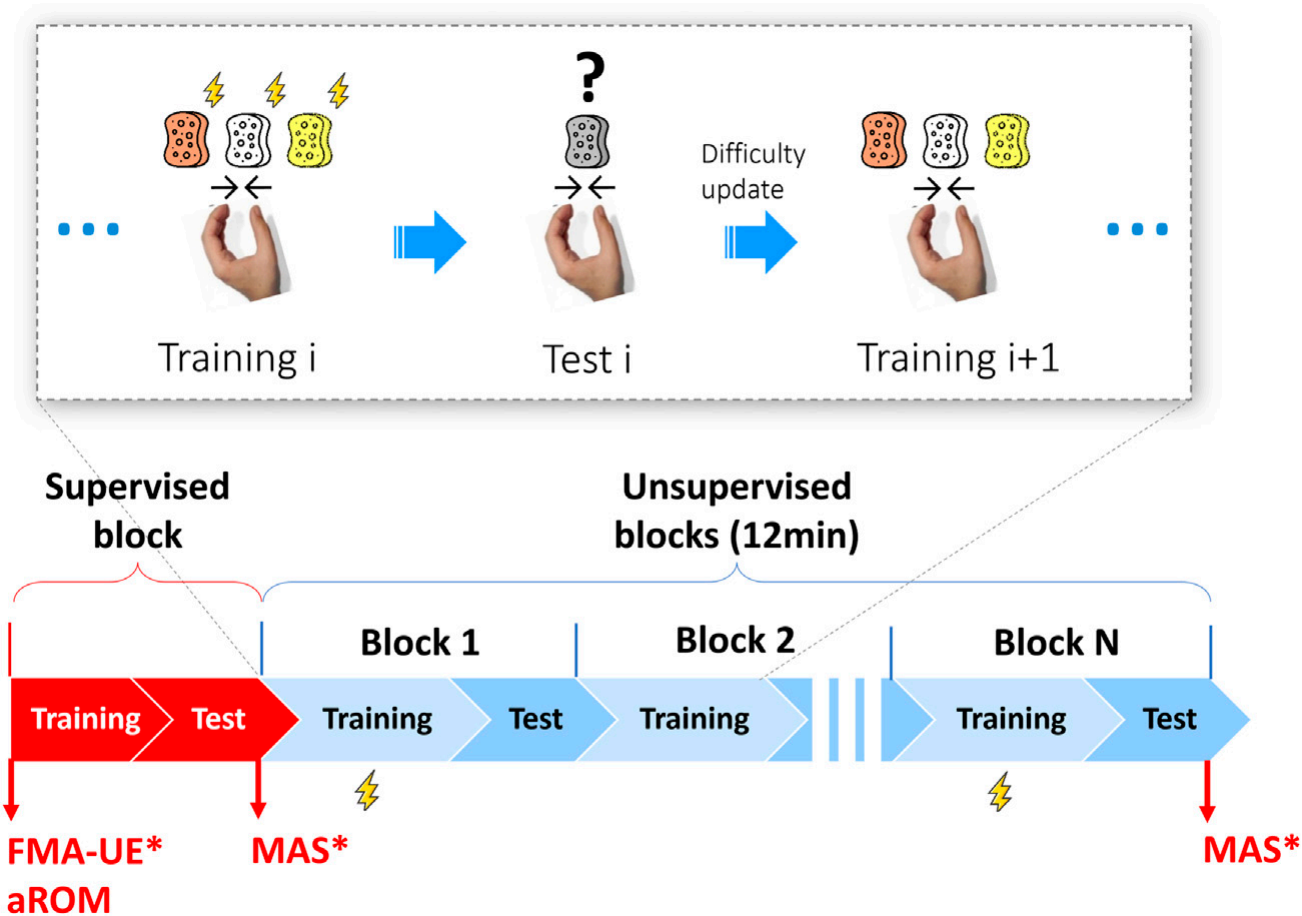
Exercise description and pilot study experimental protocol. Top: detail of the exercise structure. A block i consists of a training phase i and a test phase i. In the training phase, the subjects have to consecutively squeeze three virtual sponges of different color rendered by the robot and memorize their stiffness. In the training phase of the blocks where the perturbations were applied, i.e., the perturbation blocks, three fast and three slow perturbations (thunderbolt icon) are applied when subjects switch form one sponge to the following and their hand is relaxed. In the test phase, a random sponge among the three previously memorized is proposed. Subjects have to squeeze it and press the color corresponding to the identified stiffness on the pushbutton keyboard. This corresponds to one therapy task repetition. The difficulty level (i.e., relative stiffness difference between sponges) is progressively adapted based on the subject performance and, at each difficulty update, a new training phase (and block) is started. Thus, each subject performs a different number of blocks (with different duration and number of task repetitions) within the same exercise duration (12 min). Bottom: The pilot study includes a supervised (familiarization) block, followed by unsupervised blocks during which the subject independently performs the exercise, while the experimenter observes the session. At the beginning of the experiment, all subjects undergo the robotic assessment of the aROM, while only stroke subjects perform the Fugl-Meyer assessment of the upper extremity (FMA-UE) and the Modified Ashworth Scale (MAS) of long finger flexors, which is repeated at the end of the last block. Perturbation blocks were designed to be at least 3 min apart, meaning that not all the training phases have perturbations (maximum three over 12 min). * = performed only for stroke subjects.

We embedded our muscle tone monitoring method into a therapy exercise inspired by the neurocognitive method proposed by Perfetti, which consists of an object identification exercise (e.g., sponge of different properties) (Perfetti and Grimaldi, 1979). This exercise was previously tested with subjects after stroke during supervised therapy (Ranzani et al., 2020). We selected this sensorimotor exercise since it requires active recruitment of the long finger flexors and should suit the ability level of subjects with spasticity (e.g., no complex multi-DOF movements). The exercise is composed of a series of exercise blocks of different difficulty, as shown in Figure 3. One exercise block consists in a training phase and a test phase. In the training phase, three virtual sponges of different colors, each associated with a different stiffness value, are displayed to the subject. The user has to squeeze the sponges one by one by pressing the finger pad of the robot and memorize their stiffness. In the time interval between the squeezing of two sponges, while the subject’s hand is relaxed, a position perturbation is applied at the two finger pads. The training phase is repeated twice (i.e., the subject explores each sponge two times), which allows to perturb the patient’s hand six times (three fast and three slow in random order). To avoid perturbing the hand too often, perturbations are only applied during the first block and repeated in subsequent blocks only if these start at least 3 min after the previous block with perturbations. This accounts for three perturbation blocks over the total exercise duration (i.e., 12 min, see Figure 3). The blocks with perturbations are defined as “perturbation blocks.” In the test phase, one of the sponges is presented to the subject inside a black box, which does not allow to see the sponge color. The subject has to squeeze the sponge, perceive its stiffness and identify it with the corresponding color using the pushbutton keyboard. This corresponds to one therapy task repetition. The first block of the exercise has the same difficulty (i.e., relative stiffness difference between sponges) for all the subjects. The difficulty is progressively updated using Parameter Estimation by Sequential Testing (PEST) (Taylor and Creelman, 1967) based on the subject’s performance within the block. Each difficulty update requires a new training phase (and block) to allow the training of a new set of sponges. Thus, the number and duration of the blocks varies for each subject within the same exercise duration.

### 2.4 Stiffness identification experiment with springs

**FIGURE 4:**
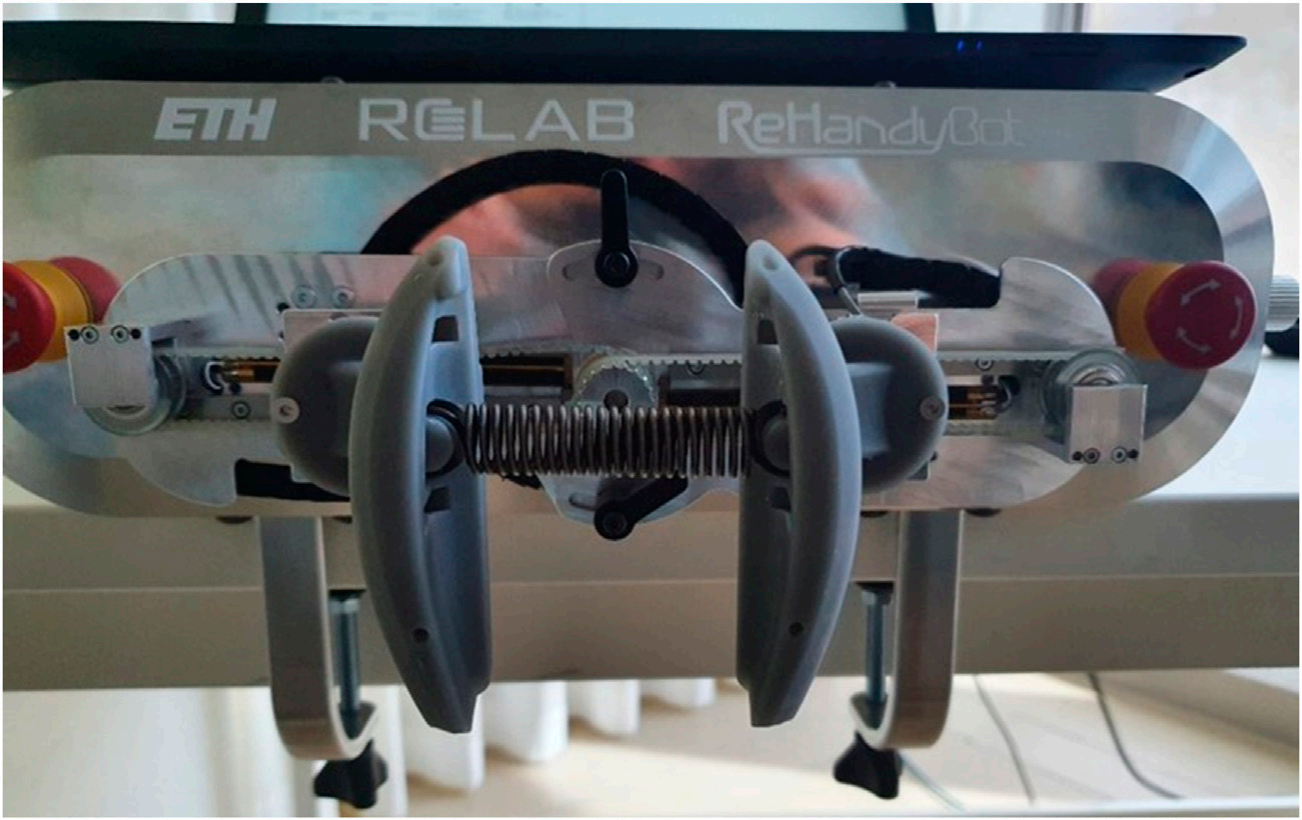
Setup for the stiffness identification experiment. Linear springs with known stiffness (0.97 or 1.27 N/mm) were applied on the inside of the finger pads through two cylindrical constraints while applying the position perturbations. This allowed to validate the performance of the perturbation-based stiffness identification.

Muscle tone can be mathematically evaluated as the change in resistance (e.g., force or stiffness) per unit change in length (e.g., Δ force/Δ displacement of the tissue) (Ganguly et al., 2021). Since the perturbation-induced force reactions at the finger pads are used to compute tone with a 20 mm displacement, a first testbench experiment was carried out to verify the reliability (i.e., measurement error) of the force (and stiffness) identification. Ten slow and ten fast perturbations were applied to two different springs with known stiffness (0.97 and 1.57 N/mm respectively), which were connected to the inside of the finger pads through two cylindrical constraints, as shown in Figure 4. These specific springs were chosen to generate reaction forces that, for a displacement of 20 mm and based on data collected during a previous clinical study (Ranzani et al., 2020), are within the range of forces typically generated by stroke patients interacting with the robot during functional therapy tasks (i.e., around 30 N).

### 2.5 Pilot study with subjects with and without spasticity in hand muscles

We conducted a pilot study in which six subjects with spasticity in the hand muscles after chronic stroke (>6 months) and ten age-matched neurologically intact subjects (i.e., >50 years old) were enrolled to participate in a single experimental session. The participants gave written informed consent, and the study was approved by the ETH Zurich Ethics Commission, Switzerland (EK 2020-N-84). Subjects with stroke were recruited through the University Hospital Zurich and included if they had a Modified Ashworth Scale (MAS) in the long finger flexors greater than or equal to one, and residual ability to lift the arm against gravity. Exclusion criteria were clinically significant concomitant diseases (i.e., severe aphasia, severe cognitive deficits, severe pain), suspected non-compliance, drug or alcohol abuse, and inability to give informed consent and understand two stage commands.

The timeline of the study protocol is reported in Figure 3. At the beginning of the experimental session, the Fugl-Meyer assessment for the upper extremity [FMA-UE, (Fugl-Meyer et al., 1975), for stroke subjects only] and the robotic assessment of the active range of motion [aROM, (Metzger et al., 2014)] of the hand during opening/closing were performed to define the ability level of the subjects and adjust exercise parameters. Subsequently, the investigator instructed the subject on how to interact with the robot (e.g., how to place, adjust and remove the hand from the finger pads) and its graphical user interface, and how to perform the exercise during one supervised block (no perturbation). After a short break, each subject had to independently perform the sponge exercise (see Section 2.3) for 12 min during simulated unsupervised blocks. During this time, the investigator sat at the back of the room, silently observed the subject’s actions and intervened only in case of risk or upon request. The stroke and unimpaired participants were tested using their impaired or dominant hand, respectively. For the stroke subjects, the MAS of the long finger flexor muscles was assessed at the beginning and at the end of the last unsupervised block, to investigate potential change in muscle tone. The MAS was chosen since it is the most widely used test for the measurement of muscle hypertonia and spasticity in research and clinical practice (Bakheit et al., 2003). An experienced physiotherapist performed all clinical assessments.

### 2.6 Outcome measures

For each perturbation *p*, independently from whether it is applied to the springs in the stiffness identification experiment or to the human hand in the pilot experiment, we report the starting position of the perturbation at the onset of the ramp, the absolute force peak *F*_*peak*_ achieved in reaction to the perturbation, and the end-point stiffness k identified at the finger pads (i.e., the estimated stiffness of the physical spring in the spring experiment or of the combination of muscle tone and finger biomechanical properties in the pilot experiment, which is the grip stiffness). These parameters allow to fully characterize muscle tone at given perturbation amplitude (i.e., 20 mm) and speed (i.e., slow and fast, with ramp times of 250 and 150 ms). All the positions and velocities are calculated with the distance in mm between the fingertip of the thumb and of the index finger.

The stiffness *k* is calculated as

$$k_{x}\left(p\right)=\frac{1}{a}\left(F_{peak}\left(p\right)-F_{base}\left(p\right)\right)$$ (1)

where *p* indicates a single perturbation, *x* is the perturbation speed (i.e., *s* = slow, *f* = fast) and *a* is the amplitude of the perturbation (i.e., 20 mm). *F*_*base*_ is the baseline grasping force before the perturbation, which is calculated as the average force over the 50 ms before the ramp onset. *F*_*peak*_ is the absolute force reaction after the perturbation (i.e., the sum of the perturbation-induced force reaction and the baseline force), which is calculated as the peak force reached between 50 ms before and 50 ms after the ramp ends. This time interval was empirically chosen based on the physiological duration of reflexes and after visual inspection of pilot data, as it is long enough to capture force changes induced by the perturbation without including voluntary reactions. The appropriateness of this empiric choice is evaluated by reporting the peak time (i.e., delta time between the perturbation onset and the maximum force peak achieved during the ramp-and-hold perturbation). *k*_*x*_ and *F*_*peak*_ are also evaluated as average over one perturbation block (i.e., average of three measurements for each perturbation block) to study their evolution over the course of the therapy session. To illustrate the human-robot interaction happening during one perturbation, Figure 2 shows one representative example of how *F*_*base*_ *, F*_*peak*_ and *k* are calculated after a fast perturbation in one stroke and one unimpaired subject (e.g., the force peak and stiffness are higher in the subject with stroke, the amplitude *a* of the ramp is constant at 20 mm independently of the exact baseline position, which can slightly vary among subjects, for instance, due to different hand sizes).

To evaluate the accuracy of the stiffness *k* identification and peak force *F*_*peak*_ measurement, in the stiffness identification experiment, the root mean square percentage error (RMSPE) is calculated between the known stiffness/force exerted by the springs and the stiffness/force measured through the robot.

### 2.7 Data analysis

In the stiffness identification experiment, the Wilcoxon rank sum test was used to verify homogeneity in stiffness identification accuracy independently on the perturbation speed.

In the pilot study, homogeneity between groups was assessed with respect to baseline characteristics and exercise dose (i.e., exercise duration, number of therapy blocks, task repetitions and therapy intensity, which is the number of task repetitions performed per time unit in the test phase), to exclude their possible influence on muscle tone, and between peak times, to evaluate if the time of perturbation-induced reflexes changes in spasticity with respect to an unimpaired population. To compare baseline characteristics, the two-sample *t*-test was used for continuous variables (i.e., age, aROM), while the Fisher’s exact test was used for dichotomous variables (i.e., gender, hand dominance, impaired hand). To assess the homogeneity between groups with respect to exercise dose, the Wilcoxon rank sum test was used for exercise duration and number of therapy blocks, while the two sample *t*-test was used for number of task repetitions and therapy intensity (i.e., number of task repetitions performed per minute during the test phase). The Wilcoxon rank sum test was used to compare the peak times between the groups.

To guarantee the assumption that the hand of the subject was at rest at the beginning of a perturbation, and therefore being able to compare our results to the MAS, all data containing obvious voluntary contractions were not included in the data analysis. In both groups, a voluntary contraction at the perturbation onset was deemed present if the *F*_*base*_ was above one standard deviation of all the baseline forces *F*_*base*_ of the subject during the exercise or if *F*_*base*_ was greater than *F*_*peak*_. However, for the scope of this analysis, to account for possible continuous increases in finger stiffness due to biomechanical reasons (e.g., due to permanent contractures), which are unrelated to voluntary muscle contraction, we previously detrended the forces with a first order fit in the participants with chronic spasticity.

The perturbation-induced peak forces were compared in a 2 × 3 aligned rank transform for non-parametric analyses of variance [ART-ANOVA, (Wobbrock et al., 2011)] (i.e., group × perturbation block, perturbation speed × perturbation block) to analyze between and within-group differences. Excluding comparisons in baseline and exercise dose, a Bonferroni correction was applied to the statistical significance level *α* = 0.05 in the analyses of the outcome measures, leading to a *p*-value for statistical significance of 0.0083.

Missing data points, due to the presence of voluntary contractions during the perturbations, were inferred by last observation carried forward or, if no former value was available, by next observation carried backward. However, if the exercise was prematurely terminated, only data up to the termination of the exercise were used, to respect the time alignment with last MAS assessment, which was performed right after the termination of the exercise.

## 3 Results

### 3.1 Stiffness identification experiment with springs

Through ten fast and ten slow perturbations, the ReHandyBot identifies spring stiffnesses with a RMSPE of 3.8% (percentage error between 2.3% and 6.9%) for the soft spring and 11.3% (percentage error between 9.8% and 12.5%) for the stiff spring. Percentage errors were not significantly different depending on the perturbation speed (Z = −0.1, *p* = 0.910). The perturbations were applied from a baseline position of 75.6 (0.6) mm and 80.1 (0.4) mm [expressed as mean (std)], and reached force peaks of 64.7 (0.4) N and 97.9 (0.3) N, respectively.

### 3.2 Participants baseline characteristics and exercise dose

All participants completed the protocol without adverse events related to the device and only two stroke subjects requested therapist intervention to reposition the hand in the finger pads during the exercise. Table 1 reports the baseline clinical and demographic characteristics. The stroke group consisted of six participants (four male, two female, age 64.3 (9.5), 141.8 (56.7) months post stroke, all right-handed), which were severely to moderately impaired (five left-impaired, one right-impaired) at the level of the upper limb in terms of FMA-UE [18.7 (9.3) out of 66 points (Woytowicz et al., 2017)] and MAS [3.5 (1.4) out of 5 points]. In terms of aROM, they were able to actively open the hand 62.0 (14.6) mm and three of them did not have any ability to actively extend their fingers above the minimum hand distance between the robot finger pads (i.e., 51 mm). The unimpaired group consisted of ten participants [two male, eight female, age 59.9 (6.1), eight right-handed and two left-handed], which were able to actively open the hand 113.7 (15.8) mm. The two groups were homogeneous in terms of gender, hand dominance and age, and were significantly different in terms of hand impaired/tested (two-tailed Fisher’s exact test, *p* = 0.035) and aROM [t (14) = 6.522, *p* < 0.0001].

**TABLE 1:** Baseline characteristics.

Category	Stroke	Unimpaired	p^a^
Gender (male, female)	4 M, 2 F	2 M, 8 F	0.118
Hand dominance (left, right)	6 R	8 R, 2 L	0.500
Impaired hand/tested hand (left, right)	1 R, 5 L	8 R, 2 L	0.035*
Age [years] [mean (std)]	64.3 (9.5)	59.9 (6.1)	0.269
aROM [mm] [mean (std)]	62.0 (14.6)	113.7 (15.8)	0.000*
Time post stroke [months] [mean (std)]	141.8 (56.7)	—	—
FMA-UE [mean (std)]	18.7 (9.3)	—	—
MAS [mean (std)]	3.5 (1.4)	—	—

^a^ *p*-values are associated with the Fisher’s exact test for categorical variables, while the two-sample *t*-test is used for continuous variables (independent samples). Abbreviations: FMA-UE, Fugl-Meyer Assessment of the upper extremity (range 0–66); MAS, modified Ashworth scale of long finger flexors (range 0–5); aROM, active range of motion. * = statistically significant result with *α* = 0.05.

In the stroke group, subjects three (male, 59 years old, 167 months post stroke, right-handed and left-impaired, FMA-UE of 15 out of 66 points, MAS 5 out of 5 points, aROM of 52.2 mm) and four (female, 55 years old, 233 months post stroke, right-handed and left-impaired, FMA-UE of 10 out of 66 points, MAS 3 out of 5 points, aROM of 87.7 mm) terminated the exercise earlier (i.e., after 6 and 7.4 min, respectively) due to slight pain at the level of the hand, which disappeared right after. Mild hand pain can be generally perceived in highly spastic hands during physical activity and quickly disappeared, and it was therefore not considered an adverse event related to the device. Due to the premature exercise termination, for subjects three and four only one and two perturbation blocks were available, respectively. In the unimpaired group, subject two (female, 62 years old, right-handed, aROM 132.5 mm) terminated the exercise after 4 min since this subject was feeling unwell on the experiment day and did not want to continue the test, and therefore had only one perturbation block.

To evaluate homogeneity in exercise dose considering all subjects, the stroke and unimpaired group performed, respectively, 10.5 (3.1) and 12.0 (3.2) min of exercise (U = 95, *p* = 0.313), 5.2 (1.9) and 6.5 (2.2) therapy blocks (U = 98, *p* = 0.161), 24.3 (11.6) and 41.2 (12.9) task repetitions [t (14) = 2.619, *p* = 0.020] with an intensity of 4.5 (1.3) and 6.6 (0.9) task repetitions per minute [t (14) = 3.718, *p* = 0.002]. Only the therapy intensity between the groups was statistically significantly different.

### 3.3 Overall group- and speed-dependency of peak times and muscle tone

Among all subjects, the perturbations were applied from a baseline position of 65.5 (5.4) mm. In the stroke group, slow and fast perturbations induced peak force reactions after 282.6 (28.7) ms and 167.4 (29.7) ms, respectively. In the unimpaired group, slow and fast perturbations induced peak force reactions after 249.7 (119.4) ms and 159.2 (87.9) ms. The difference in peak time between the stroke and unimpaired group after slow (Z = 2.5, *p* = 0.013) and fast perturbations (Z = 1.5, *p* = 0.128) was not statistically significant after Bonferroni correction.

Tables 2, 3 report the overall muscle tone estimates (i.e., considering all blocks) in terms of force peak and stiffness results after fast and slow perturbations in the stroke and unimpaired group. The force peaks after perturbation were statistically significantly different between the groups after both slow [t (127) = 5.649, *p* < 0.0001] and fast perturbations [t (127) = 7.101, *p* < 0.0001]. No statistically significant speed-dependency in the force peaks was present in both the stroke [t (88) = −2.506, *p* = 0.014] and unimpaired group (Z = −1.5, *p* = 0.122). Similarly, the stiffness results were statistically significantly different between the groups both after slow [t (127) = 3.876, *p* < 0.001] and after fast perturbations [t (127) = 4.725, *p* < 0.0001]. No statistically significant speed-dependency in the stiffness results was present in both the stroke [t (88) = −2.510, *p* = 0.014] and unimpaired group (Z = −1.9, *p* = 0.056).

**TABLE 2:** Force peak (*F*_*peak*_) results considering all the fast or slow perturbations throughout the exercise in the stroke and unimpaired groups.

	Stroke	Unimpaired	p^a^
Fast perturbations [mean(std)]	13.7 (5.6) N	6.8 (5.1) N	<0.0001*
Slow perturbations [mean(std)]	10.7 (5.6) N	5.8 (4.2) N	<0.0001*
p^a^	0.014	0.122	

^a^ *p*-values are associated with the two-sample *t*-test across the row/column. Only the fast-slow comparison in the unimpaired group is performed with the Wilcoxon rank sum test. * = statistically significant result with *α* = 0.0083.

**TABLE 3:** Stiffness (*k*) results considering all the fast or slow perturbations throughout the exercise in the stroke and unimpaired groups.

	Stroke	Unimpaired	p^a^
Fast perturbations [mean (std)]	0.49 (0.21) N/mm	0.30 (0.21) N/mm	<0.0001*
Slow perturbations [mean (std)]	0.38 (0.19) N/mm	0.25 (0.17) N/mm	<0.001*
p^a^	0.014	0.056	

^a^ *p*-values are associated with the two-sample *t*-test across the row/column. Only the fast-slow comparison in the unimpaired group is performed with the Wilcoxon rank sum test. * = statistically significant result with *α* = 0.0083.

### 3.4 Muscle tone evolution over exercise time

**FIGURE 5:**
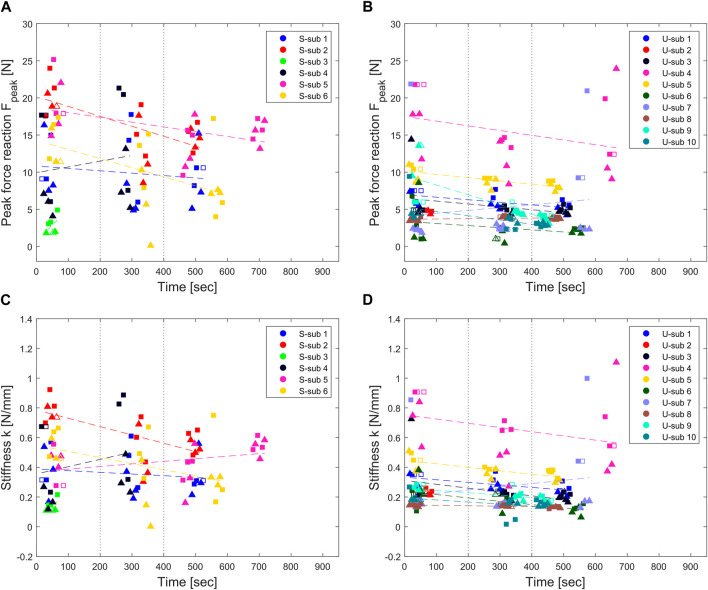
Peak force and stiffness results of individual perturbations over time in the stroke [**(A,C)**, respectively] and unimpaired group [**(B,D)**, respectively]. Triangular and squared markers represent slow and fast perturbations, respectively. The markers are empty when the perturbation was applied during a voluntary contraction and was thus replaced by the previous or next perturbation [8.8 (4.5)% and 17.2 (7.6)% of the perturbations in the stroke and unimpaired group, respectively]. Vertical dotted lines represent the division between time clusters matching the perturbation blocks. Colored dashed lines represent the line fit of the perturbation results over time for the individual subjects.

Individual force peak and stiffness results are plotted for all the perturbations over exercise time in Figure 5. Dashed lines represent the line fit of single subject’s perturbation results over time. Four out of six stroke participants and nine out of ten unimpaired participants showed a decreasing muscle tone trend over time. The average steepness of the peak force line fit and stiffness line fit are 0.00 (0.02) N/s and 0.00 (0.00) N/(mm s) in the stroke group, and −0.01 (0.01) N/s and 0.00 (0.00) N/(mm s) in the unimpaired group, respectively. Vertical dotted lines represent the division in three time-clusters matching the three perturbation blocks (i.e., blocks, divided by at least 3 min, in which the perturbations were applied).

**FIGURE 6:**
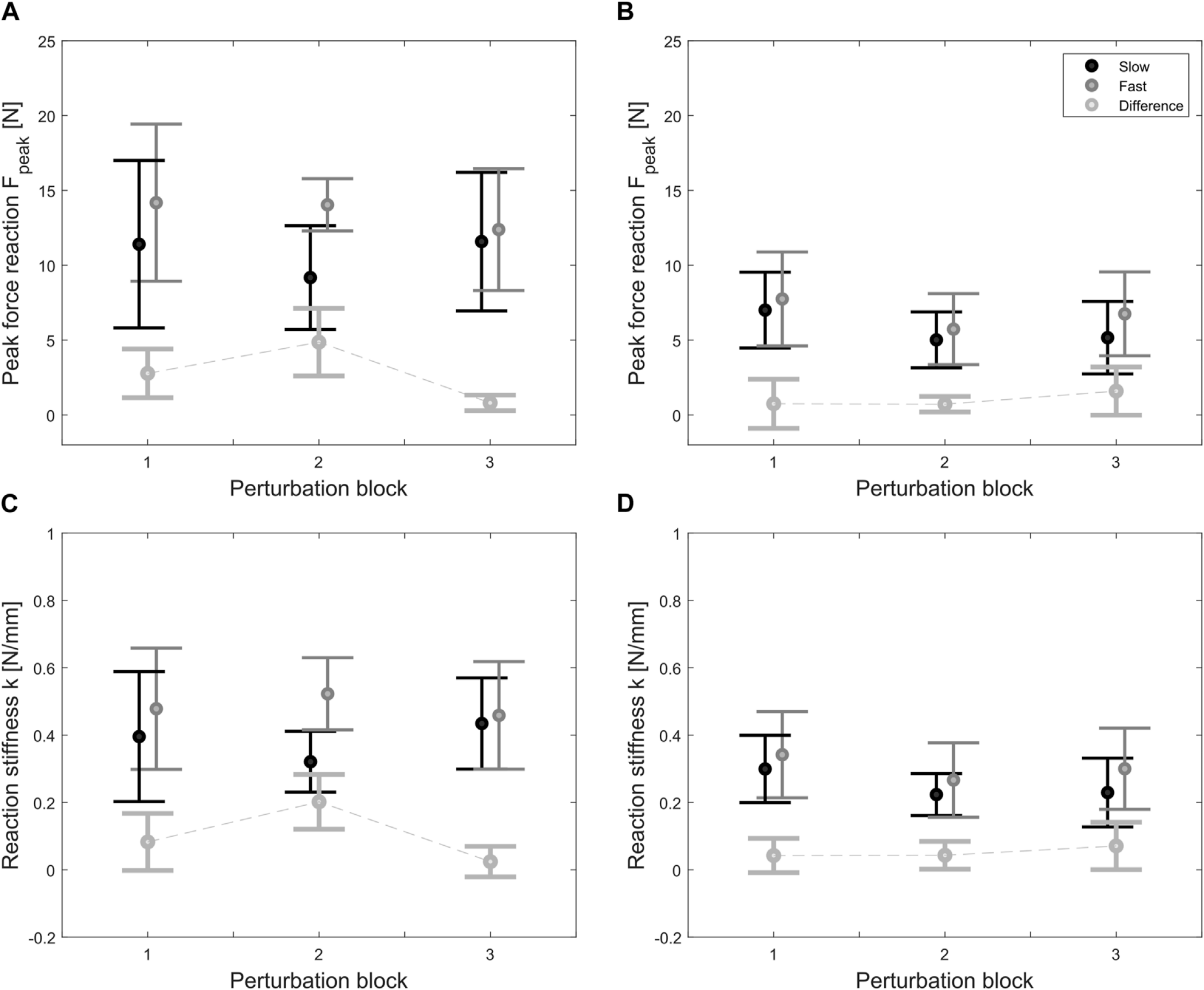
Average peak force and stiffness results at the three different perturbation blocks in the stroke [**(A,C)** respectively] and unimpaired group [**(B,D)**, respectively]. Black and gray lines represent the results after slow and fast ramp-and-hold perturbations (20 mm, 150 and 250 ms). The light gray dotted line is the difference between fast and slow results, which represents the trend in speed-dependency (i.e., zero corresponds to no speed-dependency) over time.

Peak force and stiffness results averaged per subject and perturbation block are shown in Figure 6 with 90% confidence intervals depending on the perturbation speed and group. Light grey boxplots represent the difference between the results after fast (gray) and slow (black) perturbations, and allow to see if there is any speed-dependency in the results (i.e., no speed-dependency would correspond to zero) and how this varies over the perturbation blocks. In the stroke group (Figure 6A), the peak forces after slow and fast perturbations start from a similar range of 11.4 (6.8) N and 14.2 (6.4) N at the first perturbation block, and remain approximately constant at 11.6 (3.9) N and 12.4 (3.5) N at the last perturbation block, reaching a maximum force peak of 21 N. In the unimpaired group (Figure 6B), peak forces after slow and fast perturbations are lower than the ones of the stroke group, corresponding to, respectively, 7.0 (4.4) N and 7.8 (5.4) N at the first perturbation block, and at 5.2 (3.9) N and 6.8 (4.5) N at the third perturbation block.

In both groups, according to ART-ANOVA, force peaks were not significantly different in terms of speed [stroke: F (1,24) = 2.741, *p* = 0.1108; unimpaired: F (1,50) = 1.276, *p* = 0.2640] and time/perturbation block [stroke: F (2,24) = 0.136, *p* = 0.8734; unimpaired: F (2,50) = 0.869, *p* = 0.4256], without any significant interaction effect between speed and perturbation block [stroke: F (2,24) = 0.655, *p* = 0.5285; unimpaired: F (2,50) = 0.079, *p* = 0.9246]. Independently on the speed, peak forces were statistically significantly different with respect to the group allocation [slow: F (1,37) = 12.841, *p* = 0.0001; fast: F (1,37) = 19.568, *p* < 0.00001] but not significantly different over perturbation blocks [slow: F (2,37) = 0.443, *p* = 0.6455; fast: F (2,37) = 0.781, *p* = 0.4652], and there was no interaction effect between perturbation block and group [slow: F (2,37) = 0.425, *p* = 0.6571; fast: F (2,37) = 0.625, *p* = 0.5410].

Regarding identified end-point stiffnesses, trends similar to the force peaks results are visible and replicate the same significance pattern at ART-ANOVA. In the stroke group (Figure 6C), the end-point stiffness after slow and fast perturbations starts from 0.40 (0.24) N/mm and 0.48 (0.22) N/mm at the first perturbation block, and remains at 0.43 (0.12) N/mm and 0.46 (0.14) N/mm. In the unimpaired group (Figure 6D), the end-point stiffnesses after slow and fast perturbations are lower at, respectively, 0.30 (0.17) N/mm and 0.34 (0.22) N/mm at the first perturbation block, and at 0.23 (0.17) N/mm and 0.30 (0.19) N/mm at the third perturbation block.

### 3.5 Comparison with modified Ashworth scale

In the stroke group, the average MAS in long finger flexors (scale 0–5) both before the beginning of the exercise and at the end of the exercise was 3.50 (1.38). Subjects one to three and five maintained the same MAS throughout the exercise (MAS = 1, 4, 5, 4, respectively). Subject four increased the MAS of one point, from three to four, while subject six decreased the MAS of one point, from four to three. Comparing MAS scores before and after the exercise with perturbation results at corresponding perturbation blocks (i.e., one and three), no correlation between MAS and force peak results after slow (*ρ* = 0.111, *p* = 0.7454) and fast perturbation (*ρ* = 0.223, *p* = 0.5092) could be found, nor between MAS and stiffness results after slow (*ρ* = 0.123, *p* = 0.7184) and fast perturbation (*ρ* = 0.242, *p* = 0.4732).

## 4 Discussion

This paper presented the development and validation of a method to objectively evaluate muscle tone/stiffness in the long finger flexors using a robotic device. This method is embedded into a meaningful rehabilitation exercise allowing for the online and unsupervised monitoring and documenting of muscle tone fluctuations during robot-assisted therapy. Muscle tone is assessed automatically using a perturbation-based force estimation method that measures, approximately every 3 min, perturbation-induced force peaks and end-point stiffness at the level of the fingertips, which can be used as an estimate of hand muscle tone and spasticity (e.g., in case speed-dependent muscle hypertonia is detected). A testbench experiment with physical springs first allowed to quantify the accuracy in stiffness identification of the method (and device). As a second step, a pilot study was conducted on severely to moderately impaired chronic stroke patients with spasticity and age-matched unimpaired subjects to determine the range of force reaction and corresponding end-point stiffness (as well as their evolution over exercise time) in patients with spasticity.

### 4.1 The method can accurately assess end-point stiffnesses

The stiffness identification experiment showed that proposed method implemented on the ReHandyBot robot can identify the end-point stiffness (and forces) of physical springs applied at the finger pads with errors of 3.8% (maximum < 7%) and 11.3% (maximum < 12.5%) for a soft and a stiff spring, respectively. The identification accuracy of the spring stiffness does not vary depending on the perturbation speed, as expected for physical springs. The stiffness estimation of the stiff spring has a higher error as high end-point forces tend to bend the metallic support on which the finger pads are mounted as well as the beam load cells, causing a small offset in the force measurement. These results are nevertheless satisfactory for two reasons. First, the reported errors in stiffness identification are lower than other devices designed for stiffness identification in human joints, which reported maximum errors between 15% and 25% (Tucker et al., 2017; Shi et al., 2020). Second, our measurements in the pilot study demonstrated that the force peaks and corresponding end-point stiffnesses achieved by unimpaired and spastic participants are below the values reported from the soft spring. Therefore, we can assume that our measurements with participants have, on average, errors below 4% coming from the device. However, other factors that cannot be captured, such as differences in finger placement on the finger pads while grasping, are still present and might contribute to generate more errors than the device itself.

### 4.2 The method captures between-groups differences

Our muscle tone estimation method could detect muscle tone differences between the unimpaired and stroke group, as demonstrated by the significant changes in overall force peaks and stiffness between the two groups. The force peaks and stiffness of approximately 12 N and 0.44 N/mm in the stroke group, and of 6.3 N and 0.28 N/mm in the unimpaired group match our previous results, where we showed that unimpaired elderly subjects above 50 years have force reactions and end-point stiffness up to 7.7 N and 0.39 N/mm probably due to increased finger stiffness (Ranzani et al., 2019), as well as other results in literature. According to Shi et al. (2020), the range of metacarpophalangeal (MCP) joint stiffness among four different studies with exoskeletal devices and 27 subjects with chronic stroke and spasticity in long finger flexors (MAS between one and four, out of five), varies between 0.09 and 1.13 Nm/rad (Kamper and Rymer, 2000; Towles et al., 2010; Brokaw et al., 2011; Shi et al., 2020). Furthermore, three studies with 17 unimpaired participants, reported a range of 0.01 to 0.21 Nm/rad (Esteki and Mansour, 1996; Kuo and Deshpande, 2012; Shi et al., 2020). Assuming an average MCP to distal interphalangeal joint distance of 5 cm (when proximal and distal interphalangeal angles are 45° and 20°, and a load applied over four fingers, like in our test scenario with the ReHandyBot) (Prudencio et al., 2014), these correspond to endpoint stiffnesses of approximately 0.07 to 0.90 N/mm in stroke subjects and 0.01 to 0.17 N/mm in unimpaired subjects. Van Doren (1998) and Hoppner et al. (2011) observed similar values for the endpoint stiffness of a 2-finger grasp (i.e., thumb and index), which range between 0.05 and 0.60 N/mm in unimpaired participants.

Our method captures speed-dependency in the results in the stroke group, but this relation was found to be not significant after Bonferroni correction. This might be due to the small perturbation amplitude (i.e., 10 mm at the fingertip per finger and, approximately, 0.2 rad at the MCP), which is less than half of what is typically used in conventional or robot-assisted tone assessments (Bohannon and Smith, 1987; Kamper and Rymer, 2000; Shi et al., 2020). However, this amplitude allows to make the perturbations almost non-noticeable (a necessary feature for embedding this into a rehabilitation exercise) and reduces the risk of overstretching for subjects with spasticity and, as in the case of this study, reduced aROM. Our perturbation speeds (i.e., 40 and 66.7 mm/s, i.e., approximately 0.8 and 1.33 rad/s at the MCP) could also limit the ability of the method to capture speed-dependency in the stretch reflexes, since they are within range of the speeds used in other robotic assessments [e.g., 0.11–5.2 rad/s (Kamper and Rymer, 2000)] but very close to each other.

### 4.3 Muscle tone profile does not significantly vary during robot-assisted therapy

The exercise could be independently used by all the subjects without adverse events in simulated unsupervised settings. Allowing stroke subjects with severe spasticity to independently perform a robot-assisted sensorimotor therapy exercise is a significant achievement, as these subjects are usually not included in the target population that could benefit from active therapy with rehabilitation devices. Nevertheless, the subjects tested in this study were then able to perform the exercise autonomously, despite their severe impairment. Only two of them stopped the exercise in advance to do mild pain at the level of the fingers, which is frequent and temporary for subjects with spasticity.

The analysis of force peaks and end-point stiffness over exercise time/blocks shows that both perturbation speeds allow to capture group differences, and that the muscle tone level does not vary significantly over the course of the exercise. There is limited consensus in literature on the effects of upper limb exercise on muscle tone. Highly intensive therapy might contribute to temporarily increase muscle tone, particularly in spastic patients, due to the increased motor activity (Bobath and Bobath, 1950; Perfetti and Grimaldi, 1979; Veerbeek et al., 2017) or the mental stress associated with intensive therapy (Cheung et al., 2015). These hypotheses led in the past to the exclusion of strengthening or high-intensity training from neurorehabilitation programs (Pak and Patten, 2008), to reduce the risk of long-term negative consequences of spasticity (e.g., pain, reduced functional ability and recovery) (Formisano et al., 2005; Kong et al., 2012). While in our pilot study we did not reach very high therapy intensity, we could still underline that an exercise involving repeated active hand movements is feasible for spastic stroke patients without negative effects on finger muscle tone. On the contrary, our subjects showed either a mildly decreasing or a steady muscle tone. This matches other studies and reviews showing that upper limb training does not have a negative effect on upper limb spasticity (Ada et al., 2006; Pak and Patten, 2008; Harris and Eng, 2010; Graef et al., 2016) or mildly reduces muscle tone and cocontraction (Bütefisch et al., 1995; Miller and Light, 1997; Lee et al., 2016). In fact, it has been suggested that short-term loosening of the joints and a muscle tone reduction may happen when the fingers are stretched more than three times (Charalambous, 2014; Levine, 2018; Shi et al., 2020), and in our exercise, in addition to the six perturbations in the perturbation blocks, the sponges have a size and a stiffness that slightly stretch the fingers of the user at each task repetition. This suggests that more than the intensity, the appropriate choice of robot-assisted exercise presented to spastic patient (i.e., therapy task and adaptation depending on their muscle tone level) is essential to prevent abnormal muscle tone increase during therapy. The outcome measures of the proposed method did not correlate with the MAS, however this is not surprising given the limited resolution and reliability of the scale, and its differences in assessment paradigm (e.g., single and lower speed, different range of motion) (Katz and Rymer, 1989; Melendez-Calderon et al., 2013).

### 4.4 Limitations and future work

Our results are limited by the relatively small sample size tested and the limited number of perturbation blocks performed in this pilot study. Thus, they should be further investigated in a larger population. It should also be noted that the proposed method cannot disentangle the contribution of (passive) biomechanical changes at the level of the fingers (e.g., muscle contractures, adhesions) and (active) neurological contributions to muscle tone (Nordin and Frankel, 2001; O’Sullivan et al., 2019). This could influence the magnitude (and speed-dependency) of our results. However, hypertonia at the level of the hand seems to be mostly neurological after stroke (Kamper et al., 2006). Our measurements could be dependent on the tested hand (i.e., impaired hand in the stroke group, dominant hand in the unimpaired group), which was significantly different between the groups. In fact, the robot we used cannot capture force asymmetries between fingers and thumb, which might be present in subjects after stroke (Towles et al., 2010). However, this asymmetry might be compensated by the symmetric motion coupling between the two finger pads in the device (Lambercy et al., 2014) and the stretch reflex coupling between the fingers and the thumb [e.g., a stretch applied to the fingers trigger a force reaction also in the thumb (Kamper et al., 2014)]. Future experiments with a larger population could investigate the effect of higher therapy dose within a single session, consider higher finger forces in the exercise and include more than one session, to further investigate the evolution of muscle tone depending on the exercise intensity over a longer time. Furthermore, different perturbation speeds (i.e., less close to each other) should be tested.

## 5 Implications and conclusion

The proposed method to monitor muscle tone, embedded into a rehabilitation exercise, opens new avenues for the use of robotic devices during unsupervised human-robot interactions, also with severely impaired subjects after stroke. Within a robot-assisted rehabilitation program, our method will allow for objective and quantitative (remote) monitoring of muscle tone changes. This will help better understanding how to optimize therapy settings [e.g., adaptation of exercises and their difficulty based on objective measures, such as muscle tone (Devittori et al., 2022)] for each patient in order to prevent pathological increases in muscle tone and maintain the safety of robot-assisted rehabilitation at high therapy doses, even in an unsupervised setting.

## Data Availability

The original contributions presented in the study are included in the article/Supplementary Material, further inquiries can be directed to the corresponding author.

## References

[B1] Ada L. Dorsch S. Canning C. G. (2006). Strengthening interventions increase strength and improve activity after stroke: A systematic review. Aust. J. Physiother. 52 (4), 241–248. 10.1016/s0004-9514(06)70003-4 17132118

[B2] Aggogeri F. Mikolajczyk T. O’Kane J. (2019). Robotics for rehabilitation of hand movement in stroke survivors. Adv. Mech. Eng. 11 (4), 168781401984192. 10.1177/1687814019841921

[B3] Artemiadis P. K. Katsiaris P. T. Liarokapis M. V. Kyriakopoulos K. J. (2010). “Human arm impedance: Characterization and modeling in 3d space,” in 2010 IEEE/RSJ International Conference on Intelligent Robots and Systems, Taipei, Taiwan, 18-22 October 2010 (IEEE), 3103–3108.

[B4] Bakheit A. Maynard V. Curnow J. Hudson N. Kodapala S. (2003). The relation between Ashworth scale scores and the excitability of the α motor neurones in patients with post-stroke muscle spasticity. J. Neurology, Neurosurg. Psychiatry 74 (5), 646–648. 10.1136/jnnp.74.5.646 PMC1738448 12700310

[B5] Bessler J. Prange-Lasonder G. B. Schaake L. Saenz J. F. Bidard C. Fassi I. (2021). Safety assessment of rehabilitation robots: A review identifying safety skills and current knowledge gaps. Front. Robotics AI 8, 602878. 10.3389/frobt.2021.602878 PMC8080797 33937345

[B83] Bobath K. Bobath B. (1950). Spastic paralysis treatment of by the use of reflex inhibition. British J Phys. Med.: Including its Application to Industry 13 (6), 121. 15414292

[B6] Bohannon R. W. Smith M. B. (1987). Interrater reliability of a modified Ashworth scale of muscle spasticity. Phys. Ther. 67 (2), 206–207. 10.1093/ptj/67.2.206 3809245

[B7] Boman K. (1971). Effect of emotional stress on spasticity and rigidity. J. psychosomatic Res. 15 (1), 107–112. 10.1016/0022-3999(71)90079-1 5576337

[B8] Brokaw E. B. Black I. Holley R. J. Lum P. S. (2011). Hand spring operated movement enhancer (HandSOME): A portable, passive hand exoskeleton for stroke rehabilitation. IEEE Trans. Neural Syst. Rehabilitation Eng. 19 (4), 391–399. 10.1109/tnsre.2011.2157705 21622079

[B9] Burgar C. G. Garber S. L. Van der Loos PhD H. M. Deborah Kenney Ms O. Van der Loos H. F. M. Kenney D. (2011). Robot-assisted upper-limb therapy in acute rehabilitation setting following stroke: Department of Veterans Affairs multisite clinical trial. J. rehabilitation Res. Dev. 48 (4), 445. 10.1682/jrrd.2010.04.0062 21674393

[B10] Burke D. Wissel J. Donnan G. A. (2013). Pathophysiology of spasticity in stroke. Neurology 80 (2), S20–S26. 10.1212/wnl.0b013e31827624a7 23319482

[B11] Bütefisch C. Hummelsheim H. Denzler P. Mauritz K.-H. (1995). Repetitive training of isolated movements improves the outcome of motor rehabilitation of the centrally paretic hand. J. neurological Sci. 130 (1), 59–68. 10.1016/0022-510x(95)00003-k 7650532

[B12] Cha Y. Arami A. (2020). Quantitative modeling of spasticity for clinical assessment, treatment and rehabilitation. Sensors 20 (18), 5046. 10.3390/s20185046 32899490 PMC7571189

[B13] Charalambous C. P. (2014). “Interrater reliability of a modified Ashworth scale of muscle spasticity,” in Classic papers in orthopaedics (London: Springer), 415–417.

[B85] Cheung J. Rancourt A. Di Poce S. Levine A. Hoang J. Ismail F. (2015). Patient-identified factors that influence spasticity in people with stroke and multiple sclerosis receiving botulinum toxin injection treatments. Physiother. Canada 67 (2), 157–166. 10.3138/ptc.2014-07 PMC4407118 25931667

[B14] Davidoff R. A. (1992). Skeletal muscle tone and the misunderstood stretch reflex. Neurology 42 (5), 951. 10.1212/wnl.42.5.951 1579249

[B15] De-la-Torre R. Oña E. D. Balaguer C. Jardón A. (2020). Robot-aided systems for improving the assessment of upper limb spasticity: A systematic review. Sensors 20 (18), 5251. 10.3390/s20185251 32937973 PMC7570987

[B16] Dehem S. Gilliaux M. Lejeune T. Detrembleur C. Galinski D. Sapin J. (2017). Assessment of upper limb spasticity in stroke patients using the robotic device REAplan. J. Rehabilitation Med. 49 (7), 565–571. 10.2340/16501977-2248 28664214

[B17] Demofonti A. Carpino G. Zollo L. Johnson M. J. (2021). Affordable robotics for upper limb stroke rehabilitation in developing countries: A systematic review. IEEE Trans. Med. Robotics Bionics 3 (1), 11–20. 10.1109/tmrb.2021.3054462

[B18] Devittori G. Ranzani R. Dinacci D. Romiti D. Califfi A. Petrillo C. (2022). “Automatic and personalized adaptation of therapy parameters for unsupervised robot-assisted rehabilitation: A pilot evaluation,” in 2022 International Conference on Rehabilitation Robotics (ICORR), Rotterdam, Netherlands, 25-29 July 2022 (IEEE), 1–6. 10.1109/ICORR55369.2022.9896527 36176083

[B19] Esteki A. Mansour J. (1996). An experimentally based nonlinear viscoelastic model of joint passive moment. J. biomechanics 29 (4), 443–450. 10.1016/0021-9290(95)00081-x 8964773

[B20] Formisano R. Pantano P. Buzzi M. G. Vinicola V. Penta F. Barbanti P. (2005). Late motor recovery is influenced by muscle tone changes after stroke. Archives Phys. Med. rehabilitation 86 (2), 308–311. 10.1016/j.apmr.2004.08.001 15706559

[B21] Fugl-Meyer A. R. Jääskö L. Leyman I. Olsson S. Steglind S. (1975). The post-stroke hemiplegic patient. 1. a method for evaluation of physical performance. Scand. J. rehabilitation Med. 7 (1), 13–31. 1135616

[B22] Ganguly J. Kulshreshtha D. Almotiri M. Jog M. (2021). Muscle tone physiology and abnormalities. Toxins 13 (4), 282. 10.3390/toxins13040282 33923397 PMC8071570

[B23] Germanotta M. Gower V. Papadopoulou D. Cruciani A. Pecchioli C. Mosca R. (2020). Reliability, validity and discriminant ability of a robotic device for finger training in patients with subacute stroke. J. NeuroEngineering Rehabilitation 17 (1), 1–10. 10.1186/s12984-019-0634-5 PMC6942416 31900169

[B24] Giang C. Pirondini E. Kinany N. Pierella C. Panarese A. Coscia M. (2020). Motor improvement estimation and task adaptation for personalized robot-aided therapy: A feasibility study. Biomed. Eng. online 19 (1), 33–25. 10.1186/s12938-020-00779-y 32410617 PMC7227346

[B25] Graef P. Michaelsen S. M. Dadalt M. L. Rodrigues D. A. Pereira F. Pagnussat A. S. (2016). Effects of functional and analytical strength training on upper-extremity activity after stroke: A randomized controlled trial. Braz. J. Phys. Ther. 20, 543–552. 10.1590/bjpt-rbf.2014.0187 27683837 PMC5176200

[B26] Guo X. Wallace R. Tan Y. Oetomo D. Klaic M. Crocher V. (2022). Technology-assisted assessment of spasticity: A systematic review. J. neuroengineering rehabilitation 19 (1), 138–217. 10.1186/s12984-022-01115-2 PMC9733065 36494721

[B27] Hammond P. Merton P. Sutton G. G. (1956). Nervous gradation of muscular contraction. Br. Med. Bull. 12 (3), 214–218. 10.1093/oxfordjournals.bmb.a069553 13364304

[B28] Harris J. E. Eng J. J. (2010). Strength training improves upper-limb function in individuals with stroke: A meta-analysis. Stroke 41 (1), 136–140. 10.1161/strokeaha.109.567438 19940277

[B29] Höppner H. Lakatos D. Urbanek H. Castellini C. van der Smagt P. (2011). “The Grasp Perturbator: Calibrating human grasp stiffness during a graded force task,” in 2011 IEEE International Conference on Robotics and Automation, Shanghai, China, 09-13 May 2011 (IEEE), 3312–3316.

[B30] Kamper D. G. Fischer H. C. Conrad M. O. Towles J. D. Rymer W. Z. Triandafilou K. M. (2014). Finger-thumb coupling contributes to exaggerated thumb flexion in stroke survivors. J. neurophysiology 111 (12), 2665–2674. 10.1152/jn.00413.2013 24671534 PMC6442660

[B31] Kamper D. G. Fischer H. C. Cruz E. G. Rymer W. Z. (2006). Weakness is the primary contributor to finger impairment in chronic stroke. Archives Phys. Med. rehabilitation 87 (9), 1262–1269. 10.1016/j.apmr.2006.05.013 16935065

[B32] Kamper D. G. Rymer W. Z. (2000). Quantitative features of the stretch response of extrinsic finger muscles in hemiparetic stroke. Muscle. Nerve Official J. Am. Assoc. Electrodiagn. Med. 23 (6), 954–961. 10.1002/(sici)1097-4598(200006)23:6<954::aid-mus17>3.0.co;2-0 10842274

[B33] Katner T. L. Kasarskis E. J. (2014). “Muscle tone,” in Encyclopedia of the neurological sciences (Second Edition). Editors Aminoff M. J. Daroff R. B. (Oxford: Academic Press), 194–196.

[B34] Katz R. T. Rymer W. Z. (1989). Spastic hypertonia: Mechanisms and measurement. Archives Phys. Med. Rehabilitation 70 (2), 144–155. 2644919

[B35] Kong K. H. Lee J. Chua K. S. (2012). Occurrence and temporal evolution of upper limb spasticity in stroke patients admitted to a rehabilitation unit. Archives Phys. Med. rehabilitation 93 (1), 143–148. 10.1016/j.apmr.2011.06.027 22200394

[B36] Kuo P.-H. Deshpande A. D. (2012). Muscle-tendon units provide limited contributions to the passive stiffness of the index finger metacarpophalangeal joint. J. biomechanics 45 (15), 2531–2538. 10.1016/j.jbiomech.2012.07.034 22959836

[B37] Lambercy O. Lehner R. Chua K. S. Wee S. K. Rajeswaran D. K. Kuah C. W. K. (2021). Neurorehabilitation from a distance: Can intelligent technology support decentralized access to quality therapy? Front. Robotics AI 8, 612415. 10.3389/frobt.2021.612415 PMC8132098 34026855

[B38] Lambercy O. Metzger J.-C. Santello M. Gassert R. (2014). A method to study precision grip control in viscoelastic force fields using a robotic gripper. IEEE Trans. Biomed. Eng. 62 (1), 39–48. 10.1109/tbme.2014.2336095 25014953

[B39] Lambercy O. Ranzani R. Gassert R. (2018). “Robot-assisted rehabilitation of hand function,” in Rehabilitation robotics (London: Elsevier), 205–225.

[B82] Lance J. W. (1980). Pathophysiology of spasticity and clinical experience with baclofen. Spasticity: Disordered Motor Cont., 185–204.

[B40] Lee K. W. Kim S. B. Lee J. H. Lee S. J. Yoo S. W. (2016). Effect of upper extremity robot-assisted exercise on spasticity in stroke patients. Ann. rehabilitation Med. 40 (6), 961–971. 10.5535/arm.2016.40.6.961 PMC5256323 28119825

[B41] Levine P. G. (2018). Stronger after stroke: Your roadmap to recovery. New York City: Springer Publishing Company.

[B42] Lo A. C. Guarino P. D. Richards L. G. Haselkorn J. K. Wittenberg G. F. Federman D. G. (2010). Robot-assisted therapy for long-term upper-limb impairment after stroke. N. Engl. J. Med. 362 (19), 1772–1783. 10.1056/nejmoa0911341 20400552 PMC5592692

[B43] McCabe J. Monkiewicz M. Holcomb J. Pundik S. Daly J. J. (2015). Comparison of robotics, functional electrical stimulation, and motor learning methods for treatment of persistent upper extremity dysfunction after stroke: A randomized controlled trial. Archives Phys. Med. rehabilitation 96 (6), 981–990. 10.1016/j.apmr.2014.10.022 25461822

[B44] Mehrholz J. Pohl M. Platz T. Kugler J. Elsner B. (2018). Electromechanical and robot‐assisted arm training for improving activities of daily living, arm function, and arm muscle strength after stroke. Cochrane Database Syst. Rev. 2015 (9), CD006876. 10.1002/14651858.cd006876.pub4 PMC6465047 26559225

[B45] Melendez-Calderon A. Piovesan D. Mussa-Ivaldi F. A. (2013). “Therapist recognition of impaired muscle groups in simulated multi-joint hypertonia,” in 2013 IEEE 13th International Conference on Rehabilitation Robotics (ICORR), Seattle, WA, USA, 24-26 June 2013, 1–6. 10.1109/ICORR.2013.6650425 PMC4498568 24187243

[B46] Metzger J.-C. Lambercy O. Califfi A. Dinacci D. Petrillo C. Rossi P. (2014). Assessment-driven selection and adaptation of exercise difficulty in robot-assisted therapy: A pilot study with a hand rehabilitation robot. J. neuroengineering rehabilitation 11 (1), 154. 10.1186/1743-0003-11-154 PMC4273449 25399249

[B47] Metzger J.-C. Lambercy O. Chapuis D. Gassert R. (2011). “Design and characterization of the ReHapticKnob, a robot for assessment and therapy of hand function,” in Intelligent Robots and Systems (IROS), 2011 IEEE/RSJ International Conference on, San Francisco, CA, USA, 25-30 September 2011 (IEEE), 3074–3080.

[B48] Miller G. J. Light K. E. (1997). Strength training in spastic hemiparesis: Should it be avoided? NeuroRehabilitation 9 (1), 17–28. 10.1016/s1053-8135(97)00011-5 24526088

[B49] Nordin M. Frankel V. H. (2001). Basic biomechanics of the musculoskeletal system. Philadelphia, US: Lippincott Williams & Wilkins.

[B50] O’Sullivan S. B. McKibben R. J. Portney L. G. (2019). “Examination of motor function: Motor control and motor learning,” in Physical Rehabilitation, 7e. Editors O'Sullivan S. B. Schmitz T. J. Fulk G. (New York, NY: F. A. Davis Company).

[B51] Pak S. Patten C. (2008). Strengthening to promote functional recovery poststroke: An evidence-based review. Top. stroke rehabilitation 15 (3), 177–199. 10.1310/tsr1503-177 18647724

[B81] Pandyan A. Gregoric M. Barnes M. Wood D. Wijck F. v. Burridge J. (2005). Spasticity: clinical perceptions, neurological realities and meaningful measurement. Disabil. Rehabil. 27 (1–2), 2–6. 15799140 10.1080/09638280400014576

[B52] Perfetti C. Grimaldi L. (1979). Rieducazione motoria dell'emiplegico. Milano: Ghedimedia.

[B53] Profeta V. L. Turvey M. T. (2018). Bernstein’s levels of movement construction: A contemporary perspective. Hum. Mov. Sci. 57, 111–133. 10.1016/j.humov.2017.11.013 29202312

[B54] Prudencio A. Morales E. García M. A. Lozano A. (2014). “Anthropometric and anthropomorphic features applied to a mechanical finger,” in International Conference on Intelligent Robotics and Applications, Guangzhou, China, December 17–December 20, 2014 (Springer), 254–265.

[B55] Pruszynski J. A. Kurtzer I. Lillicrap T. P. Scott S. H. (2009). Temporal evolution of “automatic gain-scaling”. J. neurophysiology 102 (2), 992–1003. 10.1152/jn.00085.2009 19439680 PMC2724331

[B56] Ranzani R. Eicher L. Viggiano F. Engelbrecht B. Held J. P. O. Lambercy O. (2021). Towards a platform for robot-assisted minimally-supervised therapy of hand function: Design and pilot usability evaluation. Front. Bioeng. Biotechnol. 9 (254), 652380. 10.3389/fbioe.2021.652380 33937218 PMC8082072

[B57] Ranzani R. Lambercy O. Metzger J.-C. Califfi A. Regazzi S. Dinacci D. (2020). Neurocognitive robot-assisted rehabilitation of hand function: A randomized control trial on motor recovery in subacute stroke. J. NeuroEngineering Rehabilitation 17 (1), 115. 10.1186/s12984-020-00746-7 PMC7444058 32831097

[B58] Ranzani R. Viggiano F. Engelbrecht B. Held J. P. Lambercy O. Gassert R. (2019). “Method for muscle tone monitoring during robot-assisted therapy of hand function: A proof of concept,” in IEEE 16th International Conference on Rehabilitation Robotics (ICORR), Toronto, ON, Canada, 24-28 June 2019 (IEEE), 957–962. 10.1109/ICORR.2019.8779454 31374753

[B59] Rodgers H. Bosomworth H. Krebs H. I. van Wijck F. Howel D. Wilson N. (2019). Robot assisted training for the upper limb after stroke (RATULS): A multicentre randomised controlled trial. Lancet 394 (10192), 51–62. 10.1016/s0140-6736(19)31055-4 31128926 PMC6620612

[B60] Saudabayev A. Rysbek Z. Khassenova R. Varol H. A. (2018). Human grasping database for activities of daily living with depth, color and kinematic data streams. Sci. data 5 (1), 180101–180113. 10.1038/sdata.2018.101 29809171 PMC5972673

[B61] Shi X. Q. Heung H. L. Tang Z. Q. Tong K. Y. Li Z. (2020). Verification of finger joint stiffness estimation method with soft robotic actuator. Front. Bioeng. Biotechnol. 8, 592637. 10.3389/fbioe.2020.592637 33392166 PMC7775510

[B62] Sivan M. Gallagher J. Makower S. Keeling D. Bhakta B. O’Connor R. J. (2014). Home-based computer assisted arm rehabilitation (hCAAR) robotic device for upper limb exercise after stroke: Results of a feasibility study in home setting. J. neuroengineering rehabilitation 11 (1), 163. 10.1186/1743-0003-11-163 PMC4280043 25495889

[B63] Tardieu G. Shentoub S. Delarue R. (1954). A la recherche d'une technique de mesure de la spasticite. Rev. Neurol. 91, 143–144. 14358132

[B64] Taylor M. Creelman C. D. (1967). PEST: Efficient estimates on probability functions. J. Acoust. Soc. Am. 41 (4A), 782–787. 10.1121/1.1910407

[B65] Towles J. D. Kamper D. G. Rymer W. Z. (2010). Lack of hypertonia in thumb muscles after stroke. J. neurophysiology 104 (4), 2139–2146. 10.1152/jn.00423.2009 20668270 PMC2957448

[B66] Tucker M. R. Moser A. Lambercy O. Sulzer J. Gassert R. (2013). “Design of a wearable perturbator for human knee impedance estimation during gait,” in 2013 IEEE 13th International Conference on Rehabilitation Robotics (ICORR), Seattle, WA, USA, 24-26 June 2013 (IEEE), 1–6. 10.1109/ICORR.2013.6650372 24187191

[B67] Tucker M. R. Shirota C. Lambercy O. Sulzer J. S. Gassert R. (2017). Design and characterization of an exoskeleton for perturbing the knee during gait. IEEE Trans. Biomed. Eng. 64 (10), 2331–2343. 10.1109/tbme.2017.2656130 28113200

[B68] van der Velden L. L. de Koff M. A. C. Ribbers G. M. Selles R. W. (2022). The diagnostic levels of evidence of instrumented devices for measuring viscoelastic joint properties and spasticity; a systematic review. J. NeuroEngineering Rehabilitation 19 (1), 16–18. 10.1186/s12984-022-00996-7 PMC8832664 35148805

[B69] Van Doren C. L. (1998). Grasp stiffness as a function of grasp force and finger span. Mot. control 2 (4), 352–378. 10.1123/mcj.2.4.352 9758886

[B84] Veerbeek J. M. Langbroek-Amersfoort A. C. van Wegen E. E. Meskers C. G. Kwakkel G. (2017). Effects of robot-assisted therapy for the upper limb after stroke: a systematic review and meta-analysis. Neurorehabil. Neural Rep. 31 (2), 107–121. 10.1177/1545968316666957 27597165

[B70] Ward N. S. Brander F. Kelly K. (2019). Intensive upper limb neurorehabilitation in chronic stroke: Outcomes from the queen square programme. J. Neurol. Neurosurg. Psychiatry 90 (5), 498–506. 10.1136/jnnp-2018-319954 30770457

[B71] Wobbrock J. O. Findlater L. Gergle D. Higgins J. J. (2011). “The aligned rank transform for nonparametric factorial analyses using only anova procedures,” in Proceedings of the SIGCHI conference on human factors in computing systems, Vancouver BC, Canada, May 7–May 12, 2011 (ACM), 143–146.

[B72] Wolf S. L. Sahu K. Bay R. C. Buchanan S. Reiss A. Linder S. (2015). The HAAPI (home arm assistance progression initiative) trial: A novel robotics delivery approach in stroke rehabilitation. Neurorehabilitation neural repair 29 (10), 958–968. 10.1177/1545968315575612 25782693 PMC4573760

[B73] Woytowicz E. J. Rietschel J. C. Goodman R. N. Conroy S. S. Sorkin J. D. Whitall J. (2017). Determining levels of upper extremity movement impairment by applying a cluster analysis to the Fugl-Meyer assessment of the upper extremity in chronic stroke. Archives Phys. Med. rehabilitation 98 (3), 456–462. 10.1016/j.apmr.2016.06.023 PMC5299057 27519928

[B74] Yang E. Lew H. L. Özçakar L. Wu C.-H. (2021). Recent advances in the treatment of spasticity: Extracorporeal shock wave therapy. J. Clin. Med. 10 (20), 4723. 10.3390/jcm10204723 34682846 PMC8539559

[B75] Zhang L. Guo S. Sun Q. (2020). Development and assist-as-needed control of an end-effector upper limb rehabilitation robot. Appl. Sci. 10 (19), 6684. 10.3390/app10196684

[B76] Zou H. Tao H. Zhou Z. Hu B. (2021). “Identification of mechanical impedance parameters of human upper limbs using mechanical perturbation method,” in International Conference on Man-Machine-Environment System Engineering, Beijing, China, October 23–October 25, 2021 (Springer), 141–147.

